# Bovine Serum Albumin Nanoparticles Enhanced the Intranasal Bioavailability of Silybin in Rats

**DOI:** 10.3390/pharmaceutics15122648

**Published:** 2023-11-21

**Authors:** Ana Paula Santos Tartari, Samila Horst Peczek, Margani Taise Fin, Jeferson Ziebarth, Christiane Schineider Machado, Rubiana Mara Mainardes

**Affiliations:** 1 Laboratory of Nanostructured Formulations, Universidade Estadual do Centro-Oeste, Alameda Élio Antonio Dalla Vecchia St., 838, Guarapuava 85040-167, Brazil; ap.tartari@hotmail.com (A.P.S.T.); samylahp@hotmail.com (S.H.P.); margani.fin@gmail.com (M.T.F.); jeferson_ziebarth@outlook.com (J.Z.); chrischineider@outlook.com (C.S.M.); 2 Department of Pharmacy, Universidade Estadual do Centro-Oeste, Alameda Élio Antonio Dalla Vecchia St., 838, Guarapuava 85040-167, Brazil

**Keywords:** silymarin, protein nanoparticles, pharmacokinetics

## Abstract

Silybin (SLB), an important flavonoid from silymarin, displays significant hepatoprotective, anticancer, antioxidant, and neuroprotective effects. However, its therapeutic efficacy is limited by its low solubility and bioavailability. To address these challenges, we engineered bovine serum albumin (BSA) nanoparticles (NP) loaded with SLB (BSA-NP/SLB) using the coacervation method. BSA-SLB NP exhibited a spherical shape, a mean size of 197 nm, a polydispersity index of 0.275, a zeta potential of −34 mV, and an entrapment efficiency of 67%. X-ray diffraction analysis indicated amorphization of SLB upon encapsulation. Formulation stability was upheld over 180 days. In vitro release assays demonstrated controlled diffusion-erosion release, with approximately 40% SLB released within 0.5 h and 100% over 12 h. Intranasal administration of BSA-NP/SLB in rats improved SLB bioavailability by fourfold compared to free SLB. These findings highlight the promising potential of intranasally administered BSA-NP/SLB as an alternative approach to enhance SLB bioavailability, paving the way for innovative therapeutic applications.

## 1. Introduction

**Figure 1:**
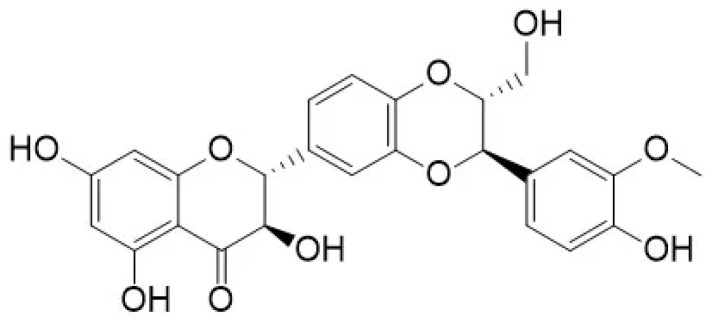
Chemical structure of Silybin.

Silybin (SLB), a polyphenolic compound (Figure 1) renowned for its antihepatotoxic properties, constitutes an active flavonoid within silymarin, an extract derived from milk thistle seeds (*Silybum marianum*) [1]. This compound holds significant hepatoprotective [2,3], anticancer [4,5,6], antioxidative [7,8], and neuroprotective effects [9,10]. Notwithstanding the substantial therapeutic potential and health-enhancing attributes intrinsic to SLB, its applicability faces certain impediments. The compound’s oral bioavailability is constrained due to its susceptibility to degradation by gastric fluids, coupled with its limited aqueous solubility. Nonetheless, efflux transporters located on the apical side of the intestinal epithelium continued to impede the absorption of silybin [1,11,12,13]. Additionally, the extensive and rapid phase II metabolism of silybin is regarded as the primary factor responsible for its diminished bioavailability. As a result, silybin is rapidly eliminated, primarily through biliary excretion [14,15]. These factors collectively limit the compound’s pharmacokinetics and its pharmacological efficacy.

Innovative avenues to enhance SLB’s bioavailability and biological effectiveness involve the strategic utilization of nanotechnology [16,17,18]. Among these strategies, polymeric nanoparticles emerge with supporting evidence. Biopolymers, encompassing polysaccharides and proteins, have garnered increasing attention for their potential in encapsulating and delivering active compounds. This interest is primarily due to their biodegradable and biocompatible properties [19,20,21]. Among these biopolymers, bovine serum albumin (BSA) stands out as a highly significant material in the realm of drug nanocarrier systems. Its appeal lies in its non-immunogenicity, non-toxicity, and biodegradability. Additionally, BSA nanoparticles offer the advantage of enhanced stability during storage and in vivo applications, and they are amenable to facile modification through covalent binding or electrostatic adsorption on their surface, thanks to their well-defined structure and high content of charged amino acids [22,23]. BSA exhibits additional merits such as cost-effectiveness, facile purification, and extensive acceptance within the biomedical sphere. The synthesis of albumin nanoparticles can be conveniently achieved through methodologies like coacervation, controlled desolvation, or emulsion formation, conducted under mild conditions. The high affinity of albumin for diverse drugs has inspired the development of albumin-based nanocarriers for drug delivery purposes [24,25].

The intranasal route of drug administration has gained significant attention in the field of pharmacology due to its potential to enhance the bioavailability of various drugs. This non-invasive route offers several advantages, primarily attributed to the extensive vascularization and large surface area of the nasal mucosa. When drugs are administered intranasally, they are absorbed directly into the bloodstream, bypassing the hepatic first-pass metabolism, which often results in higher bioavailability. This can be particularly advantageous for drugs with low oral bioavailability, as well as for patients who have difficulty swallowing or require rapid onset of action. As a result, this approach holds promise for improving therapeutic outcomes and enhancing patient compliance [26,27,28].

In the present investigation, the synthesis of BSA nanoparticles loaded with SLB (BSA-NP/SLB) is undertaken. These nanoparticles underwent characterization and assessment with a focus on bioavailability evaluation after a single intranasal administration in rats.

## 2. Materials and Methods

### 2.1. Materials

Silybin (SLB, >98% purity), bovine serum albumin (BSA, >96% purity), Glutaraldehyde, Naringin, and polyethylene glycol (PEG, Mw 2000) were purchased from Sigma-Aldrich (St. Louis, MO, USA). Absolute ethanol was purchased from Biotec^®^ (São Paulo, Brazil). HPLC-grade acetonitrile was purchased from J.T. Baker (Phillipsburg, NJ, USA). Purified water was obtained using a Milli-Q Plus system (Millipore, Burlington, MA, USA) with a conductivity of 18 MΩ.

### 2.2. Preparation of Bovine Serum Albumin Nanoparticles Containing Silybin (BSA-NP/SLB)

The synthesis of BSA-NP/SLB was achieved utilizing the coacervation technique, as described by Fonseca et al., (2017) [29], with some modifications. Initially, a solution was prepared by dissolving 100 mg of Bovine Serum Albumin (BSA) and 5 mg of Polyethylene Glycol (PEG) in 2 mL of ultrapure water, followed by thorough agitation until complete dissolution. The solution’s pH was adjusted to 9. Subsequently, a solution containing 10 mg of Silybin (SLB), dissolved in 6 mL of ethanol, was added dropwise at a controlled rate of 6 mL/min into the prepared solution under continuous stirring at 500 rpm until the onset of turbidity. To crosslink the resultant nanoparticles (NP), 50 μL of 4% glutaraldehyde was introduced, and the resulting dispersion was then transferred to microtubes for subsequent incubation. The microtubes underwent agitation within a shaker, maintained at 200 rpm and 25 °C, over a 24-h period. Following the incubation, the samples were subjected to ultracentrifugation at 15,500 rpm for 30 min at 25 °C. The supernatant was collected for subsequent analyses, while the precipitate was re-dispersed in water.

### 2.3. Physicochemical Characterization

#### 2.3.1. Particle Size and Zeta Potential Analysis

The average diameter and polydispersity index (PDI) of the particles were determined using dynamic light scattering (DLS) via the BIC 90 plus instrument from Brookhaven Instruments Corp. Zeta potential measurements were conducted employing the ZetaSizer device (ZS—Malvern^®^, Malvern, UK).

#### 2.3.2. Morphological Examination

Morphological analysis of BSA-NP/SLB was executed through scanning electron microscopy (SEM) employing the MIRA3 LM instrument from Tescan Orsay Holding, Czech Republic. SEM images were acquired at magnifications of 20,000 times.

#### 2.3.3. X-ray Diffraction (XRD) and Fourier Transform Infrared Spectroscopy (FTIR) Analysis

XRD analysis was carried out using the X-D2 PHASER diffractometer (Bruker^®^, Billerica, MA, USA), equipped with Cu Kα radiation (λ = 1.5418 Å). The scan encompassed an angular range of 2θ between 5° and 60°, with a scanning rate of 5°/min and a step size of 0.02°. FTIR analysis was performed with samples prepared as pellets containing 1% loading in potassium bromide (KBr) using the PerkinElmer^®^ (Waltham, MA, USA) FTIR spectrometer.

#### 2.3.4. Determining Entrapment Efficiency

The quantification of the SLB encapsulated within BSA-NP was indirectly determined via HPLC, following established chromatographic conditions. Analysis was performed utilizing an HPLC system equipped with a Waters^®^ (Milford, MA, USA) 2695 Alliance and a PDA Photodiode Array Detector 2998. Chromatographic conditions consisted of ultrapure water acidified with 0.05% acetic acid and acetonitrile (50:50, *v*/*v*) as the mobile phase, with a C18 column (Xterra Waters^®^ (Milford, MA, USA), 250 × 4.6 mm, 5 μm), an injection flow rate of 1.0 mL/min, an injection volume of 20 μL, a column temperature set at 30 °C, and a detection wavelength of 280 nm. The entrapment efficiency (EE) was calculated using Equation (1):

EE (%) = ((SLBi − SLBs)/SLBi) × 100(1)

whereas initial SLBi represents the initial quantity of the SLB incorporated into the NP, and SLBs refers to the portion of the SLB that remains unincorporated in the supernatant of the NP.

### 2.4. In Vitro Release Assay

In vitro release assays were conducted using a Franz-type vertical diffusion cell system (Hanson^®^, Chatsworth, CA, USA). The release medium (7 mL) consisted of a phosphate buffered saline (PBS) solution (50 mM, pH = 7.4) with 5% polysorbate 80 to maintain sink conditions. The system was kept under continuous stirring at 300 rpm and 37 °C. BSA-NP/SLB formulations containing approximately 1.5 mg of SLB were deposited onto 0.45 μm nitrocellulose membranes. Sample collection occurred at predetermined intervals (0.5, 1, 2, 4, 8, 10, 12, 24, and 48 h), with aliquots of the release medium collected and replaced. The collected samples were filtered through a 0.45 μm membrane for subsequent HPLC analysis. To decipher the release mechanism, data were analyzed using KinetDS^®^ 3.0 software (Aleksander Mendyk, GNU GPLv3 license, 2007), applying mathematical models including zero-order, first-order, second-order, and third-order models, as well as the Higuchi, Weibull, Hixson-Crowell, and Baker-Lonsdale models. The release exponent “*n*” was calculated according to the Korsmeyer-Peppas model.

### 2.5. Stability Assessment

Stability evaluation of BSA-NP/SLB encompassed monitoring physicochemical parameters such as mean particle diameter, PDI, zeta potential, and encapsulated drug content. Samples were prepared and stored at −4 °C for 6 months, with regular thawing and subsequent centrifugation at 15,500 rpm for 15 min. 

### 2.6. Pharmacokinetic Study

#### 2.6.1. UPLC-MS/MS Analysis

Ultra-performance liquid chromatography (UPLC), coupled with a triple quadrupole mass spectrometer (XEVO-TQD, Waters^®^, Milford, MA, USA) equipped with a Z sprayTM electrospray ionization source (Waters^®^, Milford, MA, USA), was employed to detect and quantify SLB levels in rat plasma after treatment. The quantification process was executed under the following chromatographic conditions: utilizing a reversed-phase C18 column (100 mm × 2.1 mm), employing a mobile phase comprised of acidified water and acidified acetonitrile, and adopting a gradient elution mode with varying proportions (90/10, 60/40, 40/60, 30/70, 90/10) at specific time intervals (0, 0.2, 0.5, 1.5, 2.0 min). The flow rate was set at 0.3 mL/min for all samples, and the total runtime encompassed 3.0 min. The retention times for SLB and the internal standard (IS), naringin, were determined to be 2.03 min and 1.45 min, respectively. An injection volume of 2 μL was employed, while the column oven temperature was maintained at 40 °C. To detect SLB, mass spectrometry was employed in negative electrospray ionization mode (ESI-). The mass spectrometry conditions incorporated a capillary voltage of 3.00 kV, a cone voltage of 60 V, an extractor voltage of 3.0 V, a source temperature of 150 °C, and a desolvation temperature of 600 °C. The quantification method relied on multiple reaction monitoring (MRM) mode, with the transitions set at *m*/*z* 481.09 → 151.90 for SLB and *m*/*z* 679.08 → 150.9 for naringin.

#### 2.6.2. Treatment

The experimental procedures adhered to ethical guidelines for animal research and received approval from the ethics committee at the Universidade Estadual do Centro-Oeste (Protocol No. 02/2022). Adult male Wistar rats weighing between 200 and 250 g were utilized for the study. These rats were housed in cages with ad libitum access to water and food and subjected to a 12-h light/dark cycle. The rats were stratified into two groups: Group A (*n* = 7), which received free SLB, and Group B (*n* = 7), which received BSA-NP/SLB. Intranasal administration was employed to deliver the formulations at a dose of 1.5 mg/kg SLB as a singular dose. Blood samples were collected from the rats at predetermined time intervals (0.5, 1, 2, 4, 8, 12, and 24 h) following administration. Subsequently, the samples (0.3 mL) were transferred to heparinized microtubes.

#### 2.6.3. Sample Preparation

Blood samples were collected via tail vein puncture and transferred to heparinized microtubes. Centrifugation at 4500 rpm at 25 °C for 10 min was conducted to extract plasma. Thereafter, liquid-liquid extraction with acetonitrile was performed. Specifically, a 50 µL plasma sample aliquot was combined with 250 μL of acetonitrile containing the IS naringin (100 ng/mL). After centrifugation at 15,000 rpm for 10 min at 25 °C, the supernatant was filtered using 0.22 µm syringe filters (Filtrilo, São Paulo, Brazil, PVDF) and transferred to an injection vial for subsequent analysis using UPLC-MS/MS.

#### 2.6.4. Data Analysis

Analysis of pharmacokinetic parameters encompassed determination of peak plasma concentration (C_max_), time to achieve peak plasma concentration (T_max_), constant of elimination (K_el_), clearance (Cl), half-life (t_1/2_), and calculation of the area under the plasma concentration-time curve (AUC_0–24h_).

### 2.7. Statistical Analysis

All experimental data were presented as mean values ± standard deviation. Statistical analyses were performed using ANOVA with a confidence level of 95%, followed by Tukey’s post-test. Statistical significance was established at *p* < 0.05 (Statistica v. 12, StatSoft Inc., Tulsa, OK, USA).

## 3. Results and Discussion

### 3.1. Preparation and Characterization of BSA-NP/SLB

BSA-NP/SLB were obtained by the coacervation technique. The concept of simple coacervation involves the alteration of protein solubility in an aqueous milieu upon the introduction of a non-solvent, typically ethanol. This leads to heightened molecular interactions while diminishing molecule-solvent associations, fostering intricate molecular assemblies [30]. In our formulation process, ethanol served as a non-solvent for BSA, driving its precipitation and enabling the solubilization and encapsulation of SLB within the polymeric matrix. The applied coacervation methodology yielded favorable outcomes, culminating in BSA-NP/SLB featuring low average diameters, high encapsulation efficiency, and negative zeta potential, effectively deviating from neutrality (Table 1). Nanoparticles were prepared with or without pH adjustment to assess their influence on the mean diameter and PDI, and the results are expressed in Table 1.

**Figure 2:**
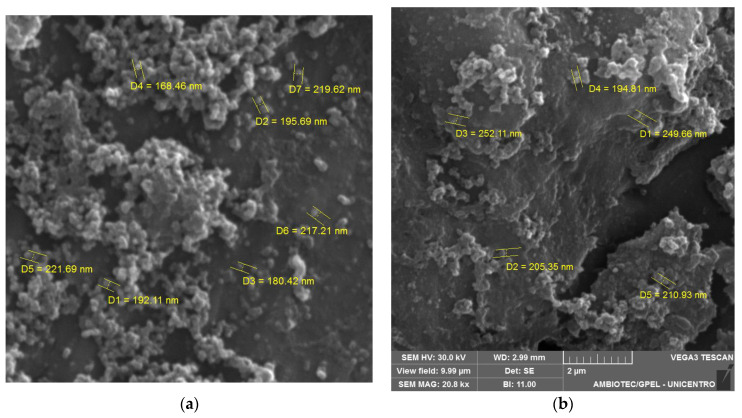
SEM images of: (**a**) BSA-NP/SLB; (**b**) unloaded BSA-NP. 20KX magnification.

Prior to adjustment, the pH of the solution was 6.4. A reduction in the mean size and PDI of the nanoparticles was observed upon pH adjustment to 9.0. BSA nanoparticles tend to aggregate around pH 5 or 6 due to their proximity to the isoelectric point of BSA (pI = 5.4). This phenomenon triggers a stark reduction in BSA solubility, prompting significant precipitation and aggregation. At pH levels exceeding 5.0, BSA molecules carry a net negative charge, leading to increased electrostatic repulsion, which in turn mitigates the coagulation of BSA molecules, ultimately resulting in the formation of smaller nanoparticles [30,31]. The negative zeta potential underscores stability within the formulations. However, the negative charge inherent to the particles impedes mucosal adhesion through electrostatic interactions, a challenge mitigated by introducing PEG in our formulation. Unloaded nanoparticles exhibited similar trends. Parameters such as size, PDI, and zeta potential hold significance in nanoparticle formulation, given their implications for stability, biodistribution, and interactions within biological systems [24]. EE% was calculated indirectly, yielding an SLB content of 67 ± 6%. The coacervation process, under favorable conditions, promotes robust drug retention via a dense polymeric network. Notably, the incorporation of crosslinking agents, enhancing thermal stability and mechanical behavior, might have contributed to the encapsulation rate [32]. SEM images evidenced the spherical shape of BSA-NP/SLB (Figure 2a) and unloaded BSA-NP (Figure 2b), with diameters similar to those measured by DLS. 

**Figure 3:**
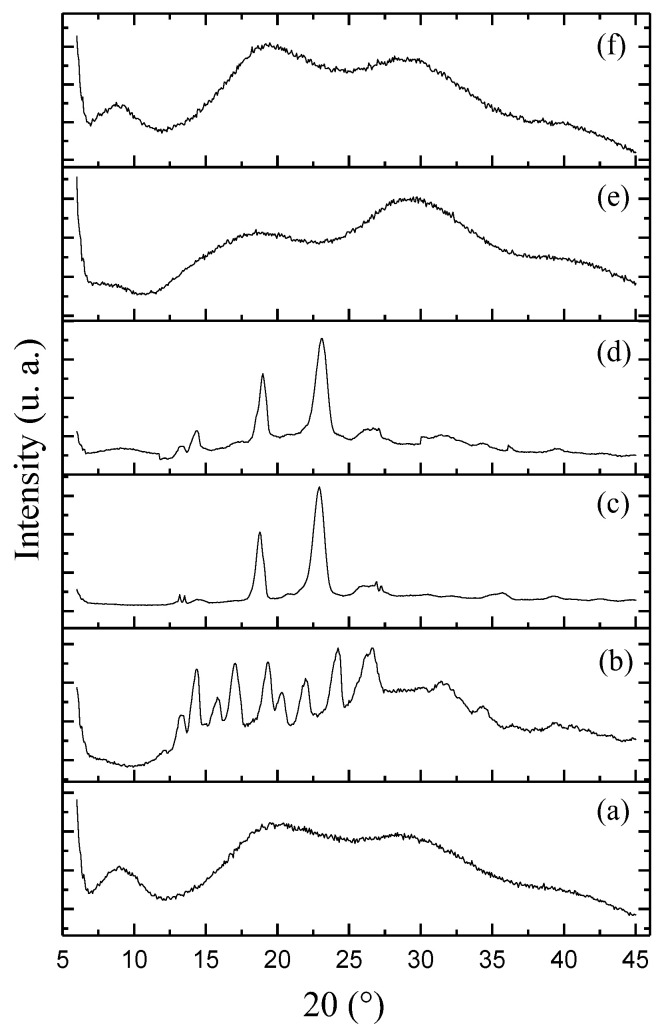
XRD curves of: (**a**) BSA; (**b**) SLB; (**c**) PEG; (**d**) physical mixture; (**e**) unloaded BSA-NP; and (**f**) BSA-NP/SLB.

In the XRD analysis, as depicted in Figure 3, the following observations were made: The XDR curve corresponding to SLB (Figure 3b) displayed prominent peaks within the range of 14° to 25°, which are indicative of crystalline characteristics. This finding aligns with the work of Pooja et al., (2014) [33] and Sahibzada et al., (2017) [17], who also noted similar crystalline patterns for SLB. In contrast, the XRD curve of BSA (Figure 3a) exhibited an amorphous pattern. The XRD of PEG (Figure 3c) exhibited peaks of lesser quantity and intensity, primarily in the range of 15° to 25°, consistent with the findings of Xiang et al., (2013) [34]. The physical mixture of the components showed an XRD related to an emicrystalline profile. The XRD of unloaded BSA-NP (Figure 3e) displayed a completely amorphous structure (Figure 3d), devoid of any discernible peaks. In addition, the XRD of BSA-NP/SLB (Figure 3f) exhibited a similar amorphous profile, lacking the characteristic crystalline peaks associated with SLB. This observation provides compelling evidence that SLB exists in a dispersed, amorphous state within the BSA-NP. These results corroborate the outcomes of other studies showing drug amorphization after encapsulation in BSA-NP [29,35].

**Figure 4:**
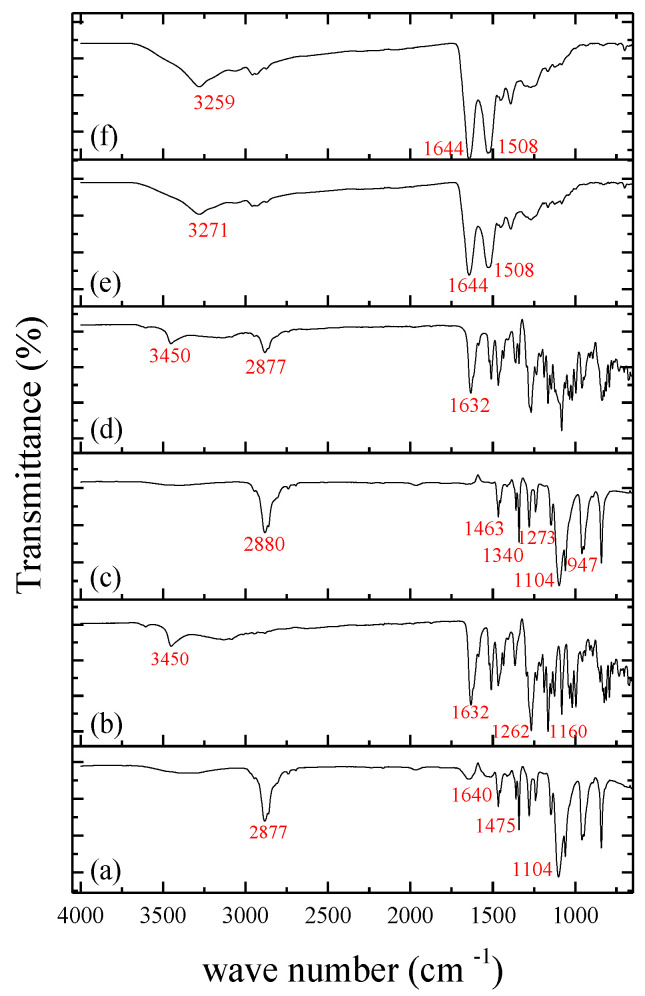
FTIR spectra of: (**a**) BSA; (**b**) SLB; (**c**) PEG; (**d**) physical mixture; (**e**) BSA-NP; and (**f**) BSA-NP/SLB.

The FTIR analysis was conducted to assess potential chemical interactions between the drug and polymers and to determine if the components retained their original characteristics after nanoencapsulation. The corresponding spectra are illustrated in Figure 4. In the pure SLB spectrum (Figure 4b), distinctive absorption bands were observed. Specifically, the band at 3452 cm^−1^ corresponds to OH stretching vibrations involving hydrogen bonds, while the band at 1632 cm^−1^ signifies C-C ring skeleton vibrations associated with aromatic stretching. Additionally, the peak at 1262 cm^−1^ relates to C-O-C stretching. These findings align with the study conducted by Pooja et al., (2014) [33]. For BSA (Figure 4a), the spectrum revealed multiple characteristic peaks. Notably, the peak at 2877 cm^−1^ is attributed to O-H elongation vibrations. The amide A band, associated with N-H elongation vibrations; amide I linked to =C-O elongation vibrations; amide II involving phase coupling of N-H bending and C-N elongation vibrations; and amide III associated with combinations of N-H in-plane bending and C-N elongation were also observed [36]. In the spectrum of PEG (Figure 4c), peaks around 2889 cm^−1^ correspond to alkyl chain elongation vibrations. The band at 1463 cm^−1^ is attributed to the folding mode of C-H groups, while the bands at 1340 and 1273 cm^−1^ are indicative of C-H torsional vibrations. The C-O elongation, C-O-H, and C-C vibrations were observed in the range of 1104–947 cm^−1^, consistent with data reported by León et al., (2017) [37]. The spectrum of the physical mixture (Figure 4d) demonstrated the absence of chemical interactions between the compounds, confirming the integrity of the polymers used. Additionally, no degradation or chemical interactions involving SLB were observed in the physical mixture. Regarding BSA-NP/SLB (Figure 4f) and unloaded BSA-NP (Figure 4e), the spectra exhibited the formation of a C=N bond, resulting from the interaction between the amino groups of the BSA and the aldehyde groups of the cross-linking agent. This bond is evident in the band around ~1644 cm^−1^ [38]. It is also suggested that the peaks at 1644 and 1508 cm^−1^ in both NPs may be influenced by a pH shift to 9.0, potentially leading to increased intensity of the amide I and II peaks, respectively [39]. 

### 3.2. In Vitro Release Assessment

**Figure 5:**
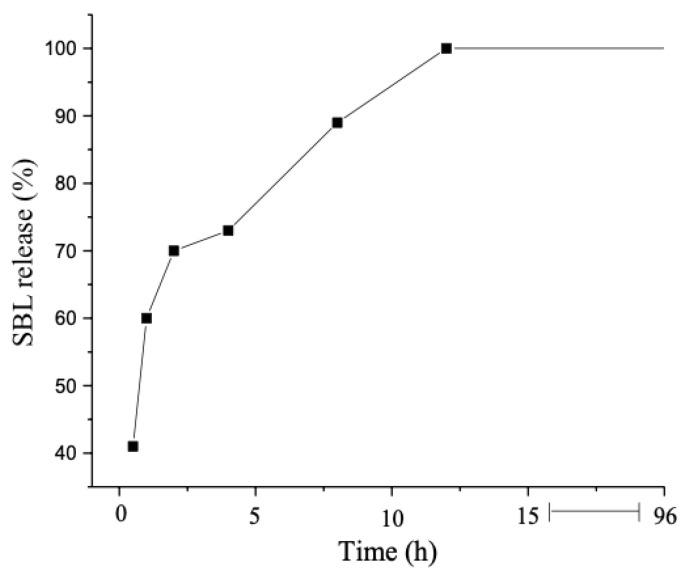
In vitro release of SLB from BSA-NP in a PBS solution (50 mM) containing 5% Tween 80 at 37 °C over 96 h.

The results of the in vitro SLB release from BSA-NP are depicted in Figure 5. BSA-NP exhibited an initial rapid release within the first few hours, with approximately 40% of the SLB being released within 0.5 h. This swift initial release of the drug, often referred to as the “burst effect”, can be attributed to the amount of SLB adsorbed or weakly bound to the surface of the NP. Upon contact with the receptor medium, the drug undergoes rapid desorption from the particle surfaces, leading to its release [29]. The release continued at a steady pace, with roughly 89% of the SLB released within the initial 8 h of the experiment and 100% of the SLB released after 12 h.

To elucidate the release kinetics of the formulation, the release profile data were subjected to mathematical analysis, leveraging the calculated correlation coefficient (r) values (Table 2). The Weibull release model was found to be the most suitable model for explaining the release data. This model is known for its adaptability, which implies drug release mechanisms encompassing dissolution, diffusion, and mixed dissolution-diffusion rate processes [40]. According to the Korsmeyer-Peppas model, the coefficient “*n*” calculated by the equation is indicative of the type of release exhibited by the matrix. Specifically, when *n* ≤ 0.43, it suggests a diffusion mechanism; *n* ≥ 0.85 indicates a matrix erosion process; and values of *n* falling between these ranges imply a combination of diffusion and erosion mechanisms [41]. In the current study, the calculated value of “*n*” was 0.47, signifying a release mechanism characterized by both diffusion and erosion.

### 3.3. Stability Assessment

**Figure 6:**
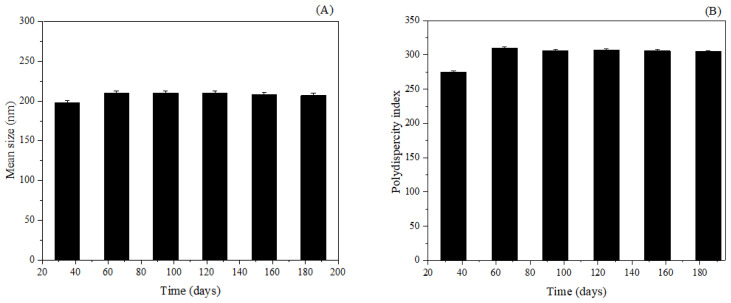

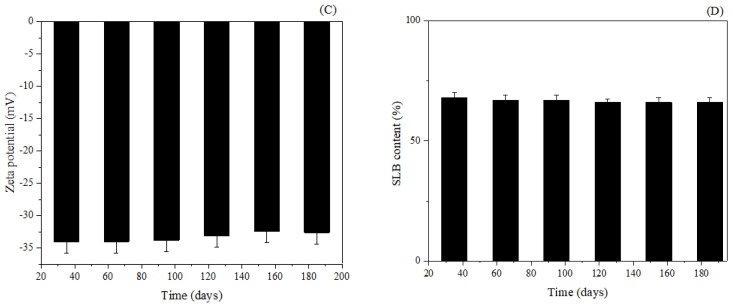
Mean size (**A**), polydispersity index (**B**), zeta potential (**C**), and SLB content (**D**) as a function of time (180 days). One-way ANOVA with Tukey post-test and α < 0.05. (*n* = 3).

During the storage period, several factors can impact the stability of NP in the medium, including aggregation leading to precipitate formation, degradation of constituents, potential inactivation of the active ingredient, and premature release of the active substance, ultimately resulting in the loss of its unique properties at the nanoscale [42]. However, over the course of 180 days, none of the assessed parameters exhibited significant alterations. Results are expressed in Figure 6.

The mean diameter of the BSA-NP/SLB displayed minor fluctuations that were statistically insignificant (*p* > 0.05) when compared to the initial diameter of approximately 198 nm (Figure 6A). This observation underscores the absence of sedimentation and aggregation within the system. Although slight oscillations in PDI values were noted, these variations did not translate into a statistically significant increase (*p* > 0.05) from the initially recorded value of 0.275 (Figure 6B). In general, PDI remained consistently below 0.3 throughout the 180-day period, ensuring that the BSA-NP/SLB maintained a favorable size distribution without any indications of aggregation or deviation from their original state.

The zeta potential, another parameter under scrutiny, exhibited no substantial changes over time (Figure 6C). Variations in this parameter could potentially arise from alterations in the Nps’ interface with the dispersing medium, potentially triggered by the dissociation of functional groups on the NP surface or the adsorption of ionic species present in the aqueous medium [29]. In this study, no significant changes were observed in the zeta potential over the duration of the study, further confirming the robust stability of the developed nanostructured system.

Lastly, the SLB content in BS-NP was quantified (Figure 6D). Throughout the evaluation period, a minor reduction in the total SLB content was noted with time. After 180 days, the percentage of drugs released was only 0.9%.

### 3.4. Pharmacokinetic Study

**Figure 7:**
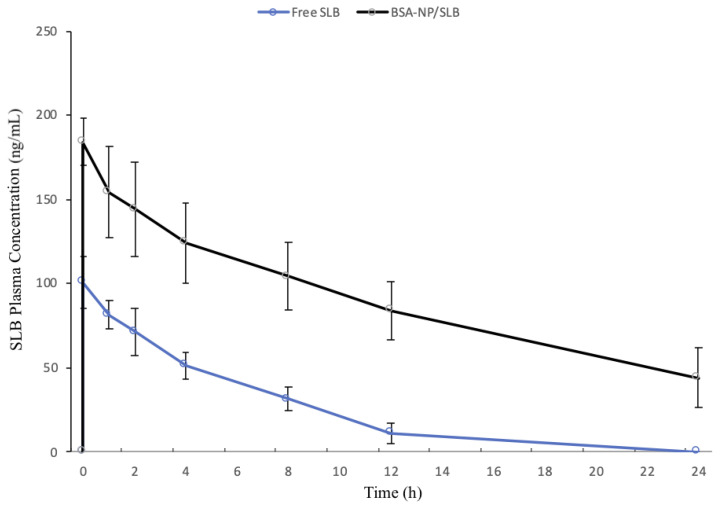
Plasma concentration-time curves of SLB obtained after single intranasal administration of BSA-NP/SLB (1.5 mg/kg of SLB) or free SLB (1.5 mg/kg) (*n* = 7).

In this study, two groups of Wistar rats were intranasally treated with an aqueous solution of SLB or BSA-NP/SLB at a dose of 1.5 mg/kg. Figure 7 displays the SLB plasma concentration-time curves after intranasal administration of different formulations. The main pharmacokinetic parameters of free SLB and BSA-NP/SLB are listed in Table 3.

Both formulations exhibited a rapid attainment of peak plasma concentration within just 0.5 h, indicating the potential of the nasal route for quick onset of action. Notably, BSA-NP/SLB outperformed free SLB, yielding a twofold higher C_max_. This indicates an enhanced absorption of SLB through BSA-NP. In contrast, free SLB was quickly cleared from the plasma, with no trace detected after 12 h, while SLB remained present in the plasma up to 24 h post-administration of BSA-NP/SLB. The nanoparticles exhibited a significantly delayed blood clearance, leading to higher SLB concentrations at later time points. The t_1/2_ of SLB increased threefold in the BSA-NP/SLB group compared to the free SLB group. Moreover, the AUC_0–24h_ value of BSA-NP/SLB was four times greater than that of the SLB solution (*p* < 0.05).

These findings suggest that the intranasal delivery of SLB through BSA-NP enhances its bioavailability in rats. This enhancement underscores the remarkable capability of nanostructured systems to modify the drug’s physicochemical properties, ultimately leading to an improved pharmacokinetic profile. Furthermore, the findings underscore the nanoparticles’ capacity to enhance SLB absorption when administered via the nasal route. Nanoparticles promote a longer retention time at the nasal mucosal surface, enhanced penetration of the drugs through the nasal epithelia, and reduced drug metabolism in the nasal cavity. This outcome aligns with the broader objective of improving drug delivery systems, particularly for compounds with low solubility or bioavailability challenges [43,44].

Silybin’s pharmacokinetics via the intranasal route present an intriguing alternative to oral administration. When administered intranasally, silybin bypasses the challenges associated with oral absorption, such as poor water solubility and extensive first-pass metabolism. The nasal route allows for direct absorption through the highly vascularized nasal mucosa, providing a more efficient and rapid entry into the bloodstream. This route can potentially lead to higher bioavailability and a faster onset of action for silybin.

The enhanced SLB absorption observed in this study carries potential clinical implications. First, it suggests that the nasal administration of SLB using BSA-NP/SLB may lead to more rapid and sustained therapeutic effects due to the higher C_max_ and prolonged presence of the drug in the bloodstream. This could result in quicker symptom relief and a longer duration of action for patients. Second, the delayed blood clearance of SLB in the BSA-NP/SLB group, as indicated by the increased t_1/2_, may reduce the frequency of dosing. Patients may benefit from less frequent administrations, leading to improved compliance and overall treatment outcomes. Moreover, the increased AUC_0–24h_ for BSA-NP/SLB compared to the SLB solution implies a higher overall drug exposure. This can be advantageous in achieving optimal therapeutic efficacy with lower doses, potentially minimizing adverse effects associated with SLB treatment. In summary, the enhanced SLB absorption through BSA-NP/SLB may translate into more effective and patient-friendly treatment options, improving the clinical management of conditions that require SLB therapy. Further clinical studies are warranted to validate these pre-clinical implications and ascertain their full potential.

## 4. Conclusions

In conclusion, the current study has successfully demonstrated that the bioavailability of SLB, a compound known for its multiple therapeutic properties, can be significantly enhanced through nanoencapsulation in BSA-NPs. The engineered BSA-NP/SLB using the coacervation method showed promising physical characteristics such as spherical shape, optimal mean size, and favorable zeta potential, which are indicative of stable nanoparticles with efficient drug entrapment. Furthermore, the amorphization of SLB post-encapsulation, as revealed by X-ray diffraction analysis, suggests an improved dissolution rate, which is often correlated with enhanced bioavailability. The controlled diffusion-erosion release pattern observed in vitro points towards the potential for achieving sustained therapeutic levels of SLB in biological systems. Most notably, the fourfold increase in bioavailability of SLB via intranasal administration of BSA-NP/SLB in a rat model is a significant finding that could revolutionize the administration of SLB and potentially other similar hydrophobic drugs. These findings collectively support the intranasal route of administration for BSA-NP/SLB nanoparticles as a highly promising alternative to traditional oral routes, which are often plagued by poor absorption and low bioavailability.

## Figures and Tables

**Table 1:** Physicochemical properties of BSA-NP with and without payload.

Sample	Mean Size ± SD (nm)	PDI ± SD	Zeta Potential + SD (mV)
BSA-NP/SLB ^1^	197 ± 17	0.275 ± 0.081	−34 ± 1
Unloaded BSA-NP ^1^	245 ± 15	0.220 ± 0.010	−32 ± 2
BSA-NP/SLB ^2^	280 ± 15	0.300 ± 0.050	−34 ± 1
Unloaded BSA-NP ^2^	301 ± 12	0.310 ± 0.012	−32 ± 1

PDI = polydispersity index; ^1^ pH adjusted to 9.0; ^2^ pH not adjusted.

**Table 2:** Mathematical models for in vitro release of SLB and their corresponding R values.

Model	R
Zero order	0.751
First order	0.426
Second order	0.145
Third order	0.790
Korsmeyer-Peppas	0.559
Higuchi	0.840
Weibull	0.937
Hickson-Crowell	0.900

**Table 3:** Some pharmacokinetic parameters obtained after a single intranasal administration of free SLB or BSA-NP/SLB in rats (*n* = 7).

Pharmacokinetic Parameters	BSA-NP/SLB	Free SLB
C_max_ (ng/mL)	184.10	101.4
T_max_ (h)	0.5	0.5
Kel (1/h)	0.0245	0.0762
Cl (L/h)	0.011	0.045
t_1/2_ (h)	28	9
AUC_0–24h_ (ng·h/mL)	921.89	218.17

## Data Availability

Data will be available on request.
